# Beyond Crisis Response: A Roundtable on Long-Term Strategies for Managing African Swine Fever

**DOI:** 10.3390/v17050604

**Published:** 2025-04-23

**Authors:** Lisa Rogoll, Katja Schulz, Jana Schulz, Jonas Brock, Hans-Hermann Thulke

**Affiliations:** 1 Institute of Epidemiology, Friedrich-Loeffler-Institut—Federal Research Institute for Animal Health (FLI), Südufer 10, 17493 Greifswald, Germany; katja.schulz@fli.de (K.S.); jana.schulz@fli.de (J.S.); 2 Department of Ecological Modelling, Helmholtz Centre for Environmental Research GmbH—UFZ, Permoserstr. 15, 04318 Leipzig, Germany; jbrock@animalhealthireland.ie (J.B.); hans.thulke@ufz.de (H.-H.T.); 3 Animal Health Ireland, 2–5 The Archways, Carrick-on-Shannon, N41 WN27 Co. Leitrim, Ireland

**Keywords:** ASF, wild boar, livestock, crisis management, world café, expert panel

## Abstract

Today, African swine fever (ASF) continues to spread in European wild boar populations, while existing management strategies respond to an animal health emergency. The current disease scenario, characterised by constant re-emergence and persistence of infection, poses a fundamental social problem for the future of ASF policy in the affected regions. A World Café workshop was organised with veterinary epidemiology experts from practice, academia and governance. The aim was to explore the problems caused by ASF in Germany for the various stakeholders and to gather perspectives for the long-term management of ASF. The panel of experts linked the unintended extension of the animal health emergency concept to the tensions between the various stakeholders and the risk of system fatigue. Sustainable management approaches need to balance rapid response to outbreaks with long-term management efforts. The experts emphasised the importance of risk-based strategies, stakeholder involvement and evidence-based policies in ASF management. The expert panel also highlighted the need for transparent communication to increase public trust and acceptance. The need for more flexible approaches requires a more open discussion about the intractable challenges posed by the long-term presence of ASF, the adequacy of existing regulations and possible visions for the future.

## 1. Introduction

African swine fever (ASF) is a viral disease invasively spreading in Eurasian wild boar populations. Spontaneous human-associated long-distance translocations result in virus jumps of hundreds of kilometres [1]. The spread of ASF infection in wild boar has led to repeated transmission of the virus between wild and domestic populations [2,3], posing an ongoing threat to national pig industries. Alongside several other European countries, Germany has been affected by ASF for several years [4]. The first case of ASF in wild boar was confirmed in September 2020 in the eastern state of Brandenburg, near the Polish border [4]. Since then, the virus has established itself in wild boar populations in several federal states, including Brandenburg, Saxony, and temporarily Mecklenburg-Western Pomerania [5]. Even though several restriction zones in the north-eastern parts of Germany have been lifted recently due to decreasing case numbers, ASF has emerged in the wild boar population of a previously unaffected area in summer 2024 in the federal states of Hesse, Rhineland-Palatinate and Baden-Wuerttemberg, where it currently continues to spread. During the past years, the disease has occasionally spread to domestic pig farms in Germany, with a total of 18 reported outbreaks in domestic pig holdings by the end of 2024.

Internationally agreed trade restrictions in the event of the presence of the ASF virus and the dramatic lethality of affected pigs make timely eradication of the virus the objective [6]. However, the continued recurrence and persistence of the infection pose a fundamental social problem for the ASF policy in the affected regions. First, an emergency approach with rigid short-term actions is confronted with a long-term problem with unclear options and unreliable outcomes. Secondly, the diverse and antagonistic interests of stakeholders cannot be easily reconciled in the long run.

The different, often incompatible stakeholder interests in relation to ASF management have been acknowledged and demonstrated repeatedly [7,8,9,10,11,12,13]. Animal health problems arising from diseases shared between livestock and native wildlife (or even humans) often impose policy measures that affect several sectors of society [14,15]. This is also the case with ASF. The primary economic impact on pig farmers and foresters is accompanied by a variety of side effects, for example, in the areas of animal welfare, psychological resilience and the need for intact landscapes for wildlife species. The sociological–participatory approach to this problem has focused on the management of a temporary crisis. Indeed, for similar animal health crises, the experiences with rapid control protocols suggest time horizons of several months to a maximum of two years [16,17]. In this context, the exceptional situation and its presumed temporary nature justify rigid legislative intervention in the field of property and property rights.

However, currently, ASF seems to be moving relentlessly away from being an emergency crisis and towards a consolidated long-term burden despite the application of intensive control measures and localised control success. This applies particularly to countries where a constant infection pressure from infected wild boar of neighbouring countries is present, e.g., due to non-harmonised measures [18]. The problem arises when the aim of elimination is continuously missed, but no alternative goal for sustainable long-term management exists. To close this gap in a sustainable way, an understanding of the motivation and conflicts of the affected stakeholders is important, and a consolidated overview of the advantages and disadvantages of basic options for action is required. In this sense, our work seeks to broaden the understanding of conflicts at the frontiers between rigid crisis management and sustainable long-term strategy development.

We approached this objective through a World Café study with an expert panel, with the aim of sharpening the views of affected stakeholders and discussing possible visions for the long-term management of ASF in wild boar.

## 2. Materials and Methods

### 2.1. Background and Participants

This study was conducted in the form of a half-day workshop in September 2023 in Germany, addressing the long-term conflict dynamics surrounding ASF in wild boar and aiming to explore diverse strategic perspectives for the long-term management of the ASF virus in Germany.

The workshop was organised alongside a scientific conference focusing on epidemiology and animal health management (“DACh-Epidemiologie Tagung 2023”). Participation in the workshop was voluntary and based on participants’ interest in the workshop topic. Most participants were already present at the conference venue due to their involvement in related scientific or professional activities, which facilitated a multidisciplinary composition of the group.

A total of 20 individuals participated and are hereafter referred to as the expert panel. The participants had a veterinary and/or epidemiological background with a variety of professional experience from academia, public sector, pig health or veterinary practice. Participants originated mainly from Germany, Austria or Switzerland.

In addition, three stakeholder representatives—each representing a key affected stakeholder group (hunters, pig farmers and disease managers)—participated in the workshop. These representatives were purposefully selected and invited to provide authentic stakeholder insights.

### 2.2. Workshop Concept and World Café Structure

At the beginning of the workshop, the three invited stakeholder representatives each gave an impulse statement from a “partisan” perspective. Each statement outlined three conflict areas or key challenges perceived as critical, assuming a long-term persistence of ASF in wild boar from their stakeholder group’s viewpoint. They were asked to explain how and why these problems arise, with the explicit aim of introducing subjective and undecided viewpoints into the discussion. Stakeholder positions were summarised, and three conflict items of each stakeholder group were derived by the facilitators and endorsed by the stakeholder representatives.

Out of these, participants selected the most contentious conflict item per stakeholder group. Each participant had one vote for each stakeholder group.

The three selected conflict items became the basis for the World Café roundtable discussions. The World Café method is a structured conversational process designed to foster open and creative dialogue around key questions. Participants rotate between small-group discussions at different tables, allowing collective knowledge-building through repeated engagement with different viewpoints [19,20,21].

In our setting, the participants were randomly assigned to three equally sized participant groups. Following the World Café method, each group rotated between three tables, each dedicated to one of the selected stakeholder-specific conflict items.

Each table discussion was moderated by external facilitators with experience in moderating group work. The discussion at each table followed a consistent three-step approach: (a) problem characterisation and convergent interpretation of the tabled issue, (b) reasoned arguments endorsing and rejecting the conflict potential, and (c) visions of potential solutions or pathways to solving the conflict. Participants were encouraged to present their perspectives and respond to the arguments of the others. After the three rotation rounds, a joint plenary session was held. The facilitators summarised the key arguments discussed at each table, and participants reflected on similarities and differences between perspectives.

### 2.3. Data Collection and Analysis

Data were collected in written form by the facilitators on flip charts during the joint discussions with the participants. Additional notes were taken by the facilitators during the plenary discussion.

The collected data were analysed using qualitative content analyses and summarised narratively for this manuscript. In the following, the key themes that emerged from the roundtable discussions with the expert panel are summarised. The themes are reported separately for each stakeholder group and along the following structure: (a) problem perception and understanding, (b) problem endorsement and rejection, and (c) raised visions of solution before a synopsis is proposed.

## 3. Results

### 3.1. Selected Conflict Items for World Café Study

Nine conflict items, three for each of the defined stakeholder groups (pig farmers, hunters and disease managers), were recorded by the facilitators and endorsed by the stakeholders (Table 1). Of these, the following conflict items were selected by the participants for roundtable discussion: the financial burden of pig farmers, hunters’ concern about ASF fences as an animal welfare risk to wildlife and disease managers’ struggle with prolonged crisis management (Table 1).

### 3.2. Pig Farmer’s Financial Burden

#### 3.2.1. Problem Perception and Understanding

At the beginning of the discussion, the experts engaged with the perception of the problem presented by the farmer’s impulse statement and developed the following understanding of the issue: The lasting occurrence of ASF in Germany causes a significant financial burden for domestic pig farmers. Firstly, there are increased costs for disease control measures, including adapting biosecurity protocols, establishing surveillance and sampling strategies and investing in infrastructure to prevent the spread of disease (e.g., additional fencing, proper disposal of feed, proper transport of animals). In addition, the implementation of biosecurity and sampling strategies requires an increase in labour and working hours, which adds to the financial burden. Overall, pork production costs have increased without any form of compensation and without a proportional adjustment of pork prices.

#### 3.2.2. Problem Endorsement and Rejection

The expert panel discussed the problem perception, collecting arguments for problem endorsement and rejection; they are summarised in Table 2.

The expert panel agreed that the occurrence of ASF is a financial challenge for pig farmers and has a significant impact on pig production and the economy. Experts mentioned that marketing opportunities for pork, especially for export, are negatively affected by international and national trade restrictions imposed to prevent the spread of disease, resulting in severe economic losses. However, it was also pointed out that local sales of pork were still possible without restrictions, providing relevant markets for pork despite international trade restrictions. Furthermore, experts discussed the possibility that ASF occurrence may lead to the disruption of production and food supply chains and that regional disparities between affected and non-affected regions could occur, which may lead to distortions of competition. Additionally, experts mentioned that the financial challenges may result in structural changes in the domestic pig industry in Germany regarding farm size, the occurrence of backyard farms and the closing of farms or business units. The uncertainty regarding the future may also lead to a loss of economic viability for pig production. In addition, the situation can pose an existential threat to farms, which is accompanied by great emotional distress for the farmers concerned. Due to reduced economic viability, the experts feared negative impacts on animal welfare and food quality, as well as reduced investments in ecological or free-ranging pig farming. On the other hand, experts argued that in the long term, food prices may adjust and result in increased income for pig farmers. There has been a perspective suggesting that the problem may be more of an emotional and psychological nature than a real economic threat. In addition, the experts pointed out that there are still several other options available to farmers for the use of their land and for commercial purposes.

#### 3.2.3. Raised Visions of Solution

To mitigate the financial burden for pig farmers, experts proposed various potential solutions combining political, consumer-focused and innovative approaches. Firstly, they emphasised the need for political consideration of the problem, urging policymakers to evaluate the impact of ASF on pig production in Germany. This involves deliberating on the necessity of subsidies to support pig farmers, exploring opportunities to create new marketing channels and allowing compensation for both direct and indirect costs incurred by pig farmers. Additionally, the adaption of the regulatory framework was proposed as a key aspect of addressing the financial burden, including changes to animal health policy. There was a call for stricter differentiation of the consequences of an ASF outbreak in domestic pigs as compared to cases in wild boar. The experts emphasised that in a situation where ASF is almost endemic in the wild boar population and only sporadic outbreaks occur in domestic pigs, it would seem appropriate to modify the control measures to reduce the impact on domestic pig holdings. In addition to political action, experts stressed the importance of consumer awareness campaigns. By raising awareness about ASF and its implications for pig production, they hope to encourage greater consumer acceptance, thereby maintaining demand for pork products despite the presence of ASF. Lastly, the experts saw the ASF situation as an opportunity for innovation in agriculture. They suggested developing new concepts for farming practices, land use and animal husbandry that prioritise animal welfare and sustainability. By embracing innovation, pig farmers may be better equipped to adapt to the challenges posed by ASF while maintaining economic viability.

### 3.3. Hunters’ Concerns About ASF Fences and Their Impact on the Welfare of Wildlife

#### 3.3.1. Problem Perception and Understanding

At the beginning of the discussion, the experts engaged with the perception of the problem presented by the hunter’s impulse statement and developed the following understanding of the issue: Wildlife fences are intended to restrict the movement of wild boar, the main host of ASF in Germany, and to protect domestic pigs from the spread of the disease. However, stakeholders, such as hunters, fear that the fences will have a negative impact on several wildlife populations and habitats by preventing the natural movement of the animals and causing animals to become trapped, injured or even killed in the fences.

#### 3.3.2. Problem Endorsement and Rejection

In the expert panel discussions, various viewpoints regarding fencing as an ASF control measure and its effects on the welfare of wild animals were raised by the expert panel (Table 3). While some experts considered the impact on animal welfare as an issue, others saw this more as an emotional debate.

The discussion showed that the main problem for the experts was the assessment of the benefits of ASF control fences. While some experts emphasised the necessity and effectiveness of the fences in controlling the spread of the disease, others pointed to significant negative impacts on wildlife populations. The main argument put forward by the opponents was that fences cause animal suffering and that any animal that dies at these barriers is a loss and is ethically questionable. They also emphasised the suppression of genetic variability by the physical barriers if they prevent natural migration and, thus, the genetic mixing of populations. Some experts saw the argument about necessary deforestation and environmental damage caused by the construction of fences as a pretext to hide the real objectives of the debate, such as the protection of property. The discussion about forest damage and the social acceptance of the fences, especially along highways, was also dismissed as subjective and emotional, as these infrastructures are socially accepted for accident prevention.

Overall, the experts emphasised the need to quantify the impact of ASF control measures (e.g., fencing) on wildlife and strike a balance between different interests.

#### 3.3.3. Raised Visions of Solution

To address the concerns of hunting practitioners about the impact of ASF fences on wildlife welfare, experts suggested combining improved communication, data collection and innovative approaches.

Firstly, the experts emphasised the need for improved communication and awareness campaigns. They argued that better information dissemination, especially at community discussions and gatherings, could reduce misunderstandings and misinformation about the purpose and necessity of ASF control fences. Educating the public on the importance of these measures and their impact on wildlife could foster greater acceptance and cooperation.

Secondly, experts highlighted the importance of collecting data on wildlife mortality related to the fences. By systematically gathering and analysing data on animals that perish at the fences, policymakers can create evidence-based assessments of the fences’ impact.

Additionally, experts suggested removing unnecessary fences that do not significantly contribute to controlling ASF spread. This could help reduce habitat fragmentation and minimise negative impacts on wildlife.

The construction of higher and more wildlife-friendly fences was also suggested. These improved fences could be designed to allow safe passage for smaller animals while effectively containing wild boar. Such modifications would help strike a balance between disease control and wildlife welfare.

### 3.4. Disease Managers’ Struggle with Crisis Management on a Prolonged Perspective

#### 3.4.1. Problem Perception and Understanding

The expert panel summarised the disease manager’s perception of the problem in his impulse statement and developed the following understanding of the issue: Disease managers are currently faced with a key paradox in crisis management strategies—the tension between the imperative for quick, decisive action (such as rapid containment) and the recognition of the possibility of an endemic course that requires sustainable and flexible long-term management strategies. This dual demand often creates cognitive dissonance among professionals as they struggle to balance immediate responses with sustainable solutions. Disparities in disease management emerge when regional approaches are compared with national ones.

#### 3.4.2. Problem Endorsement and Rejection

The discussion centred on the challenges and considerations surrounding the long-term management of ASF. The collected arguments are summarised in Table 4.

First, the expert panel deemed the current short-term emergency approach as unsustainable, mainly due to resource limitations, public resistance and potential ineffectiveness over time. The persistent infection pressure from neighbouring countries, where ASF remains a prevalent issue, was identified as a factor exacerbating the challenge. The shortage of resources—human, material, infrastructure and financial—was seen as a pressing issue requiring a change in strategy goals. The uncertainty surrounding the long-term practicability of the current emergency approach and the possible system fatigue in their implementation underpinned the need for reconsideration of the strategic approaches. However, the experts expressed uncertainty regarding the formulation of a comprehensive long-term strategy. They recognised the challenges associated with achieving a consensus between the different interests of the various stakeholders involved. Concerns about possible sabotage or boycott of measures, as well as the lack of public acceptance, added layers of complexity to the problem. Legal constraints were noted as a barrier to implementing useful strategy changes. Lastly, the high expectations placed on all stakeholders and the recognition that economic swine production might become unviable under current conditions emphasised the urgency of addressing these challenges.

A few points were raised during the discussion on problem rejection. Experts acknowledged the lack of direct health impacts for humans that may inadvertently downplay the urgency of adopting a prolonged perspective as large parts of society remain unaffected by ASF. Similarly, arguments framing ASF primarily as an economic issue affecting a small target group or citing a lack of motivation for sustained efforts could be interpreted as rejecting the need for a long-term strategy by implying a complete cessation of measures. Furthermore, it was mentioned that resource needs may be unpredictable. Experts pointed out that the reasoning behind the rejection arguments overlooks the multifaceted nature of crises such as ASF and the need for more holistic strategies. Overall, it is important to distinguish between questioning the urgency of a long-term strategy and advocating the abandonment of all measures.

#### 3.4.3. Raised Visions of Solution

In addressing the challenge of ASF disease management, particularly in the context of a prolonged crises, the expert panel has discussed a range of considerations.

There was a consensus on the importance of flexibility in the fight against ASF. Disease managers need to be able to reassess and reallocate resources in the long term based on the evolving situation. This requires a dynamic approach that can quickly adapt to changing circumstances to ensure efforts remain focused and effective. In this context, the development of risk-based strategies was discussed as a component of effective long-term ASF management. By identifying and prioritising high-risk areas and populations, resources could be allocated more efficiently, maximising their impact.

The expert panel underlined the need for a flexible regulatory framework that can adapt to changes in the epidemiological landscape. The discussion highlighted the need to speed up legislative changes to allow timely adaptation of regulations to evolving circumstances. At the same time, it was emphasised that the regulatory framework should provide for the possibility of dealing with unforeseen events.

The expert panel discussed the need to promote acceptance of the long-term effort to control ASF. Raising awareness and understanding among the general public and policy makers of the transition from a crisis situation to a long-term problem was discussed as an important approach. This includes communicating the need to move from a short-term emergency approach to a sustainable long-term management strategy.

In addition to these overarching principles, experts discussed specific strategies to enhance disease management practices. These included the development of harmonised processes for managing ASF in wild boar populations, promoting interdisciplinary communication and untangling federal structures to streamline decision-making processes.

The development of a vaccine, solid data collection for evidence-based decision-making and the creation of separate legal frameworks for domestic and wild boar populations were identified as important visions for solutions. These measures were viewed as not only enhancing the efficacy of disease control efforts but also helping to increase public trust and acceptance of long-term interventions.

In summary, addressing the challenges posed by prolonged ASF crises requires a multifaceted and adaptive approach. By embracing readiness for change, developing risk-based strategies, facilitating legal changes, fostering acceptance of prolonged efforts and supporting (vaccine) research, disease managers might be able to navigate the ASF crises more effectively.

### 3.5. Synopsis of Perspectives for Long-Term Management of ASF Control

Four key elements of future perspectives for the long-term management of ASF control were brought up after the roundtable discussions. These are strategic adjustments for control strategies, awareness raising, research initiatives and regulatory changes.

Experts highlighted the need to adjust control strategies to the current situation. They propose to achieve that by simplifying federal structures to streamline decision-making processes and the development of standardised processes for managing ASF to improve effectiveness. Furthermore, the implementation of risk-based strategies and flexible allocation of resources to prioritise high-risk areas was put forward.

Raising awareness was identified as another crucial element for long-term ASF management. Engaging political decision-makers to understand the long-term challenges of ASF faced by stakeholders is vital for securing sustained support and compliance. Consumer awareness campaigns to support pig marketing and targeted campaigns to raise awareness among farmers and other stakeholders to improve compliance were suggested. Additionally, promoting interdisciplinary communication and collaboration was also emphasised for effective ASF management.

Research initiatives with a focus on comprehensive data collection and analysis to understand the full impact of ASF were deemed essential. The development of innovative concepts for animal husbandry and land use was highlighted, alongside the critical need for continued efforts regarding vaccine development.

Amending the regulatory framework to reduce the impact of outbreaks in either the domestic pig or the wild boar population on the respective other population and ensuring flexibility to accommodate evolving outbreaks was seen as a promising way forward. Additionally, implementing subsidies and compensation mechanisms was advised to support farmers and encourage compliance.

The diversity of these suggested long-term perspectives reflects the complexity of the ASF situation and the need for adaptive, interdisciplinary approaches. These perspectives also raise critical questions about the current direction of ASF management.

## 4. Discussion

African swine fever (ASF) remains a critical threat to the pork industry in Germany and across Europe [22]. The perspectives gathered in this manuscript highlight both the urgency and the challenges of developing sustainable long-term strategies. The emergence of new cases, particularly in Sweden, and in previously unaffected regions in Germany (Hesse, Rhineland-Palatine and Baden-Wuerttemberg), or the prolonged occurrence of the infection in northern Italy [22], underline the dynamic nature of the disease and the continuing challenge it poses for effective long-term management [13,23].

In this study, a World Café workshop brought together an expert panel with a background in veterinary epidemiology from practice, academia, and governance to explore the challenges of prolonged ASF management for affected stakeholders and consolidate future perspectives. The primary goal of the workshop was not to propose definitive solutions but to reflect on the range of opinions and uncertainties that currently shape the discourse (Table 5). While the workshop included impulse statements from invited stakeholder representatives, the subsequent discussions were conducted solely among the expert participants. This may have introduced certain limitations, such as a relatively homogeneous expert composition, potential authority bias from the impulse statements and a limited sample size. Nevertheless, the structured method allowed for a nuanced exploration of conflicting perspectives, and the professional diversity and field experience of the participants added depth and practical relevance to the interpretation of stakeholder concerns [24].

The format of the workshop enabled an open discussion of topics and aspects that are still unresolved or often appear to be politically insurmountable hurdles in the usually more interest-oriented debates. In the discovery discussion, it became clear which aspects represent key arguments in the professional and political debate. Interestingly, the minutes also reveal the main barriers to action that hinder the efficient implementation of change. In the integrative overview, the participants identified four key policy-relevant fields of action, which are discussed below in reflection of the existing literature. These are the need to adapt ASF control objectives and strategies to the current situation, to raise awareness of the multilateral concerns and issues of ASF management, to fill knowledge gaps with scientific and systematic evidence to promote efficient policies and to undertake political efforts to amend inappropriate regulations.

The outcome of the roundtable discussion underlines the need for a strategic reassessment of the emergency approach, including possible objectives of the action [25]. Experts acknowledged that the current approach may not be sustainable in the long term and that system fatigue could set in if measures are not adapted to changing circumstances. While the short-term emergency approach is characterised by rapid containment of outbreaks to prevent further spread by using resource-intensive, rigid measures, the long-term management strategy should focus on sustainable management and prevention.

It has already been described that the ASF epidemic has had an impact on the European pig industry, mainly due to the restrictions imposed by legislation in the event of an outbreak [6]. Experts have discussed the possibility of adapting restrictions in a way to disentangle the impact of cases in wild boar from outbreaks in domestic pig farms. The disease is maintained within the wild boar population in Germany, with only sporadic transmission to domestic pig farms; hence, the workshop outcome is consistent with the viewpoints of other affected stakeholders [5,7]. Therefore, the recent Commission Implementing Regulation (EU) 2023/594, repealing the Implementing Regulation (EU) 2021/605, already allows for derogations to approve animals/products to be moved within infected countries. However, it is extremely difficult to separate the wild and domestic sectors from a legal perspective, as the risk of transmission between domestic pigs and wild boar is always present in both directions [26]. Nevertheless, experts have questioned whether the current European goal of eradicating ASF is still appropriate, given the current situation.

The discussion emphasised the need for scientific and evidence-based elements to strengthen future approaches to ASF management. Cost–benefit analyses of control measures, such as fencing, were identified as important tools for optimising resource allocation while still missing largely in the ASF literature. Similar to the discussion in this study, the perceived benefits and drawbacks of using fences in ASF management have been discussed in several studies by affected stakeholders [7,24,27,28,29]. Fencing has proven to be an important control tool for managing focal ASF outbreaks in the European context [30], e.g., in Belgium [31]. However, several studies have pointed out that fences are cost-intensive, require ongoing maintenance, and may disrupt gene flow in wild boar populations [32,33]. While fencing continues to play a central role in ASF control strategies, comprehensive data collection on animal losses at fences and other ecological consequences remains scarce. A comprehensive analysis, including long-term epidemiological, ecological and economic aspects, was considered crucial to inform decision-making and refine intervention policies. Panel experts reiterated the need to continue research towards vaccine development. Consistently, hunters or farmers have expressed high hopes for the development of a vaccine against ASF in former studies [28], even though realistically, a vaccine against ASF will most likely not be available in the short term [34]. Another research gap concerned possible innovations in the principles of animal husbandry and production, which could even generally reduce the risk of spreading infectious diseases such as ASF in pigs.

Public acceptance was highlighted by the expert panel as a critical factor for transitioning from a short-term emergency approach to a sustainable, long-term management strategy. To foster public acceptance, strategies that address concerns and encourage cooperation from the broader public are required. Transparent and effective communication and the adoption of evidence-based decision-making are crucial to increase public trust and acceptance [35,36].

In conclusion, the challenges posed by the prolonged ASF crisis and possible visions for the future need to be discussed openly and faced with a multifaceted and adaptive approach. This study highlights the need for a shift from reactive, short-term measures to sustainable, long-term strategies for managing ASF. To achieve this, further research into cost–benefit analyses of control measures is crucial. Additionally, prioritising scientific evidence to inform policies and fostering public acceptance through transparent communication are key factors. By recognising multilateral stakeholder concerns and aligning strategies with both scientific evidence and practical considerations, the ASF control strategy could become more effective and sustainable in meeting future challenges.

## Figures and Tables

**Table 1:** The nine conflict items derived by the facilitators from the stakeholder impulse statements and endorsed by the stakeholders. Numbers in brackets show the results of the participants’ voting for topic selection (one vote per stakeholder group and participant).

Pig Farmer	Hunter	Disease Manager
Psychological stress (8)	Loss of income and wild game value (5)	Prevention (5)
Financial burden (10)	ASF fences and their impact on welfare of wildlife (15)	External influences (3)
Increased workload (2)	Encroachment of property rights (0)	Crisis management in a longer-term perspective (12)

**Table 2:** Arguments and perspectives on pig farmers’ financial burden that were collected during the roundtable discussion at the pig farmer’s table.

Problem Endorsement	Problem Rejection
Reduced options for marketing of pork	Local sales remain possible without restrictions
Loss of export markets	Long-term adjustment of food prices may increase income
Disruption of production and food supply chains	Rather an emotional or psychological burden than an economic problem
Structural changes in domestic pig holdings	Possibilities for alternative land use and commercial purposes available
Negative impact on food production standard and animal welfare	
Loss of economic viability of pork production	
Regional disparities cause competition distortions	
Existential threat to farms	

**Table 3:** Arguments and perspectives on hunters’ concerns about the impact of ASF fences and their impact on the welfare of wildlife that were collected during the roundtable discussion.

Problem Endorsement	Problem Rejection
Unclear usefulness of fences	Lack of quantitative data: No relation to how many animals die at fences
Restriction of genetic variation	Subjective assessment: Wildlife car accident vs. fence accident
Forest damage—deforestation	Many fences along highways: No social discussion about it
Animal suffering at fences is real	Animal welfare is a pretext: General rejection of measures
Hunter’s code of honour: Careful handling of life and nature	

**Table 4:** Arguments and perspectives on disease managers’ struggle with prolonged crisis management that were collected during the roundtable discussion.

Problem Endorsement	Problem Rejection
Strategy impacts public (e.g., fences, access bans)	Society unaffected (health-wise)
Persistent ASF pressure from neighbouring country	Issue primarily economic (small target group)
Resource shortage demands strategy shift	Limited motivation for long-term commitment
Long-term practicability of emergency approach uncertain	Resource needs unpredictable
What should the long-term strategy look like?	
System fatigue in implementing emergency approach	
Potential sabotage or boycott of measures	
Measures lack public acceptance	
Legal constraints hinder strategy change	
Lack of long-term ASF combat experience	
High expectations placed on all stakeholders	
Economic swine production unviable	

**Table 5:** Comparison of objectives, measures, characteristics and foci of the current short-term emergency approach as opposed to the vision of a sustainable long-term management strategy.

	Current Short-Term Emergency Approach	Long-Term Management Strategy
Objective	Rapid containment of ASF outbreaks and prevention of further spread	Prevention of ASF outbreaks using resource-conserving and sustainable measures
Measures	Immediate quarantine Culling of infected stocks Emergency plans	Independent regulations for domestic pigs and wild boars with diverging aims of activities Flexible regulations to adapt to new outbreak situations Promotion of reliable scientific evidence
Characteristics	Reactive Resource intensive Often less sustainable policies	Proactive/preventive Long-term orientated Evidence-oriented policies
Focus	Acute crisis solving	Building resilience in the domestic pig population Reducing the impact on the wild boar population

## Data Availability

The original contributions presented in this study are included in the article. Further inquiries can be directed to the corresponding author.
